# Advanced iron-overload cardiomyopathy in a genetic murine model is rescued by resveratrol therapy

**DOI:** 10.1042/BSR20171302

**Published:** 2018-01-10

**Authors:** Subhash K. Das, Pavel Zhabyeyev, Ratnadeep Basu, Vaibhav B. Patel, Jason R.B. Dyck, Zamaneh Kassiri, Gavin Y. Oudit

**Affiliations:** 1 Division of Cardiology, Department of Medicine, University of Alberta, Edmonton, Canada; 2 Mazankowski Alberta Heart Institute, Edmonton, Canada; 3 Cardiovascular Research Centre, Faculty of Medicine and Dentistry, University of Alberta, Edmonton, Canada; 4 Department of Physiology, University of Alberta, Edmonton, Canada; 5 Departments of Pediatrics and Pharmacology, University of Alberta, Edmonton, Canada

**Keywords:** cardiomyopathy, fibrosis, hemochromatosis, heart failure, iron overload, oxidative stress

## Abstract

Iron-overload cardiomyopathy is prevalent on a worldwide basis and is a major comorbidity in patients with genetic hemochromatosis and secondary iron overload. Therapies are limited in part due to lack of a valid preclinical model, which recapitulates advanced iron-overload cardiomyopathy. Male hemojuvelin (HJV) knockout (HJVKO) mice, which lack HJV, a bone morphogenetic co-receptor protein required for hepcidin expression and systemic iron homeostasis, were fed a high-iron diet starting at 4 weeks of age for a duration of 1 year. Aged HJVKO mice in response to iron overload showed increased myocardial iron deposition and mortality coupled with oxidative stress and myocardial fibrosis culminating in advanced iron-overload cardiomyopathy. In a parallel group, iron-overloaded HJVKO mice received resveratrol (240 mg/day) at 9 months of age until 1 year of age. Echocardiography and invasive pressure–volume (PV) loop analyses revealed a complete normalization of iron-overload mediated diastolic and systolic dysfunction in response to resveratrol therapy. In addition, myocardial sarcoplasmic reticulum Ca^2+^ ATPase (SERCa2a) levels were reduced in iron-overloaded hearts and resveratrol therapy restored SERCa2a levels and suppressed up-regulation of the sodium–calcium exchanger (NCX1). Further, iron-mediated oxidative stress and myocardial fibrosis were suppressed by resveratrol treatment with concomitant activation of the p-Akt and p-AMP-activated protein kinase (AMPK) signaling pathways. A combination of ageing and high-iron diet in male HJVKO mice results in a valid preclinical model that recapitulates iron-overload cardiomyopathy in humans. Resveratrol therapy resulted in normalization of cardiac function demonstrating that resveratrol represents a feasible therapeutic intervention to reduce the burden of iron-overload cardiomyopathy.

## Introduction

Hereditary (genetic) hemochromatosis and secondary iron-overload disorders are prevalent on an international scale [1–3]. Iron-overload cardiomyopathy results from prolonged exposure to excess iron [4,5]. Juvenile hemochromatosis, also known as type 2 primary hemochromatosis, is a genetic iron metabolic disorder associated with a progressive increase in iron stores due to a mutation in hemojuvelin (HJV), a bone morphogenetic co-receptor protein required for hepcidin expression [6–8]. Hepcidin is a major regulator of systemic iron homeostasis and prevents excess gastrointestinal iron absorption [1,9]. Type 2 primary hemochromatosis associated with reduced hepcidin expression results in increased gastrointestinal iron absorption leading to systemic iron overload [10]. Importantly, aggressive iron overload associated with juvenile hemochromatosis results in an early-onset form of iron-overload cardiomyopathy characterized by heart failure and arrhythmias [11–14].

In iron-overloaded conditions, iron enters the cardiomyocytes through the l-type Ca^2+^ channels resulting in increased myocardial iron deposition [15–17]. Excess cardiac iron is highly toxic and leads to free radical formation via the Fenton reaction [18,19] leading to myocardial oxidative stress, a key pathogenic mechanism of iron-overload cardiomyopathy [4,16,19]. Progressive myocardial iron accumulation is associated with diastolic dysfunction at an early stage and progresses to an end-stage dilated cardiomyopathy [4,5,15,20]. However, a preclinical model recapitulating iron-overload cardiomyopathy in humans is lacking, which serves as a major obstacle in elucidating the pathophysiology and discovery of novel therapeutics for heart failure related to iron overload.

Recently, we established that HJV knockout (HJVKO) murine model develops diastolic dysfunction, but not systolic dysfunction at the age of 6 months (end point) in response to 5 months of high-iron diet [20]. Diastolic dysfunction was accompanied by oxidative stress, fibrosis, and, in the case of wild-type (WT; injected iron dextran), by elevated sodium–calcium exchanger (NCX1) protein levels and reduced SERCa2a levels [20]. However, relatively short exposure to iron diet left a possibility that iron injury might be not severe enough to reach injury levels similar to the ones experienced by patients with unmanaged primary hemochromatosis when the patients reach overt iron-overload cardiomyopathy phenotype (at the age of 40–60 years). Therefore, in the present study, we sought to improve and better characterize the HJVKO murine model. To do so, the administration of iron (via food intake) was extended up to 1 year of age to ensure sufficient time for iron injury to develop and, thus, establish a preclinical model of iron-overload cardiomyopathy that can simulate development of systolic dysfunction in later stages of the disease. Using this improved and more adequate model, we explored the therapeutic effects of resveratrol in prevention and possible reversion of development of iron-overload cardiomyopathy. Our results suggest that myocardial oxidative stress, fibrosis, and Ca^2+^ cycling defects are linked with the diastolic and systolic dysfunction observed at the advanced stage of iron overload. Resveratrol supplementation reduces iron-overload mediated myocardial oxidative stress and reverts adverse remodeling (reducing fibrosis and normalizing expression of NCX1 and SERCa2a in comparison with previously reported 6-month-old data point) [20]. Based on this, we propose that resveratrol can be a potential therapeutic intervention to reduce and/or revert progression of iron-overload cardiomyopathy at the advanced stage of iron overload.

## Materials and methods

### Experimental animal protocols

Male HJVKO mice (*HVJ^−/−^*) (kindly provided by Dr Nancy C. Andrews, Duke University) were bred in-house at the University of Alberta Health Sciences Laboratory Animal Services housing facility. All experiments were performed in accordance with University of Alberta institutional guidelines, which conformed to guidelines published by the Canadian Council on Animal Care and the Guide for the Care and Use of Laboratory Animals published by the United States National Institutes of Health (revised 2011). We used an advanced iron-overload protocol by feeding 4-week-old HJVKO mice with high-iron diet [21] (Prolab® RMH 3000 with iron 380 parts per million (ppm)) until they were 1 year old. We determined the therapeutic effects of resveratrol on the 1-year-old iron-overloaded HJVKO mice by administering resveratrol daily via oral gavage (240 mg/kg/day) for 3 months starting at 9 months of age [20,22–24]. Resveratrol was dissolved in 5.4% ethanol/corn oil and the placebo control group received 5.4% ethanol/corn oil.

### Echocardiography and invasive hemodynamic analysis

Transthoracic echocardiography was performed with the Vevo 2100 high resolution imaging system equipped with a 30-MHz transducer and using 0.8% isoflurane [20,25–27]. Pressure–volume (PV) loop analysis was done by using 1.2F Scisense catheter connected to an amplifier (TCP-500 Scisense Inc.) as previously described [20,27,28]. Following baseline PV measurements, transient inferior vena cava occlusion was performed to obtain the alteration in venous return to derive the end-diastolic PV relationship; transient infrarenal aorta occlusion was used to derive the end-systolic PV relationship.

### Body composition

Body composition (fat mass, lean mass, free water, and total water) was assessed by using an Echo MRI-900 (Echo Medical Systems, Houston, TX), as described previously [26].

### Histology

Mice were anesthetized, and the hearts arrested in diastole by using 15 mM KCl, fixed in 10% buffered formalin, and embedded in paraffin. Five-micrometer thick sections were stained with Prussian Blue, Picrosirius Red (PSR), and Masson trichrome stain for morphometric analyses as described previously [15,16,20,27,29]. Iron depositions were visualized as blue depositions using bright-field microscope. Myocardial fibrosis was evaluated by using PSR staining followed by visualization using an Olympus IX81 microscope and image analysis using MetaMorph software [20,27].

### Immunofluorescence

Immunofluorescence was performed on 5-μm thick formalin-fixed and OCT-embedded heart sections. Briefly, formalin-fixed paraffin embedded sections were deparaffinized, followed by antigen retrieval and blocking with blocking buffer (1% BSA in 1× PBS) for 1 h [20]. Similarly, OCT-embedded sections were fixed with 4% paraformaldehyde for 20 min and rehydrated in 1× PBS for 30 min. Sections were then incubated with primary antibody against rat anti-mouse neutrophil (Serotec), rat anti-mouse F4/80 (Serotec), mouse anti-nitrotyrosine (anti-NT) (Santa Cruz Biotechnology), mouse anti-4-hydroxynonenal (anti-4-HNE) (Abcam), overnight in a humidified chamber at 4°C. Sections were incubated with different fluorophore-conjugated secondary antibodies (Invitrogen, U.S.A.) as described recently [20].

### Measurement of lipid peroxidation and glutathione levels

The levels of malondialdehyde (MDA), an indicator of lipid peroxidation, were measured in myocardial tissue (100–150 mg) by using a commercially available kit (Bioxytech, MDA-586TM assay, Oxis International Inc., Foster City, CA). Myocardial GSH and GSSG levels were measured as described recently [20,29].

### Tissue iron levels

Twenty-milligram frozen tissues from left ventricle were subjected to inductive coupled plasma resonance MS to quantitate tissue iron level in the Trace Metals Laboratory, London, Western Ontario. The samples were analyzed in triplicate and the average values were used [15,20].

### Taqman real-time PCR

mRNA expression levels were evaluated using Taqman real-time PCR (see Supplementary Table S1 for primers and probes). Total RNA was extracted from flash frozen left ventricular (LV) tissue by using TRIzol RNA extraction method [20]. One microgram of RNA was subjected to reverse transcription to synthesize cDNA. Samples were loaded in triplicate and the data were analyzed by LightCycler® 480 system from Roche.

### Western blot analysis

Western blot analysis was performed on flash frozen LV tissue samples as previously described [20,26,27]. Briefly, we extracted protein from LV tissues and performed immunoblotting for various proteins using the following primary antibodies: sarcoplasmic reticulum Ca^2+^ ATPase (SERCa2a), NCX1 (Thermo Scientific), Akt-P^Ser473^, Akt-P^Thr308^, Total Akt (Cell Signaling), AMP-activated protein kinase (AMPK)-P^Thr172^, and total AMPK (Cell Signaling) and subsequently incubated with HRP-conjugated secondary antibodies, respectively.

### Statistical analysis

All data are presented as mean ± S.E.M. and statistical analyses were performed using the SPSS Statistics software (version 23). The non-parametric log-rank test was used for the survival analysis. Effects of iron diet and resveratrol treatment were evaluated using parametric testing, one-way ANOVA followed by multiple comparison testing using the Tukey’s test. Histological data (PSR, 4-HNE, and NT stainings) were assessed using non-parametric Kruskal–Wallis test followed by the Mann–Whitney U test with Bonferroni correction. *P* values <0.05 were considered significant.

## Results

### Increased myocardial iron accumulation in an aged murine model of iron overload

**Figure 1:**
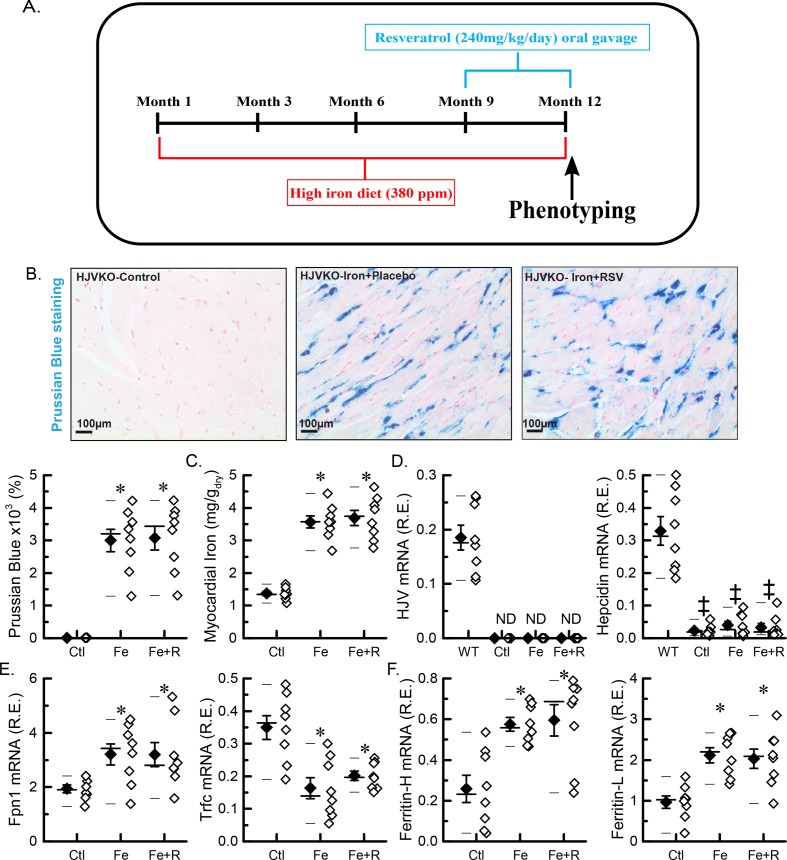
Myocardial iron overload in an aged murine model of iron-overload cardiomyopathy (**A**) Schematic representation of the study design. (**B**) Representative images for Prussian Blue staining with quantitation (*n*=8: four hearts; two sections per heart). (**C**) Total myocardial tissue iron levels (*n*=8 hearts). (**D**) Expression of iron metabolic genes: HJV and hepcidin *(Hamp*). (**E**) Expression of iron transporting genes: ferroportin (FPN1) and transferrin receptor 1 (Trfc). (**F**) Expression of iron storage genes: ferritin light (L) chain and ferritin heavy (H) chain. *n*=8 (hearts) for gene expression analysis (D–F); *P<0.05 compared with the Ctl group. ^‡^*P*<0.05 compared with the WT standard group. Abbreviations: Ctl, HJV control; Fe, iron diet; Fe + R, iron diet + resveratrol; ND, not detected.

We established an experimental paradigm whereby ageing and iron overload were coupled in HJVKO mice and utilized this model to determine the therapeutic potential of resveratrol by treating these mice for the last 3 months of the 1-year high-iron diet (Figure 1A). The myocardial iron deposition was significantly increased as shown by Prussian Blue histological staining and quantitation (Figure 1B), which was confirmed by inductive coupled plasma resonance spectroscopy (Figure 1C). The myocardial expression of HJV, a co-receptor for hepcidin expression, was defective in comparison with WT tissue (a positive control) confirming that HJVKO acts as a proper knockout. Expression of ferroportin increased and expression of hepcidin decreased due to iron supplementation and was not affected by introduction of resveratrol (Figure 1D), suggesting that resveratrol does not affect dietary iron intake. The expression of transferrin receptor was decreased (Figure 1D), and expressions of ferritins (heavy and light chains) were increased (Figure 1E), which are consistent with myocardial iron overload. The degree of the overload was not altered by resveratrol (Figure 1C,E,F). Assessment of body composition by echo MRI revealed normal body composition and fasting blood glucose in all experimental groups (Supplementary Figure S1). Importantly, resveratrol treatment did not affect myocardial iron deposition or expression of iron-regulatory genes (Figure 1A–E). These molecular changes confirmed significant age-dependent myocardial iron overload.

### Resveratrol therapy rescued the advanced iron-overload cardiomyopathy and increased survival

**Figure 2:**
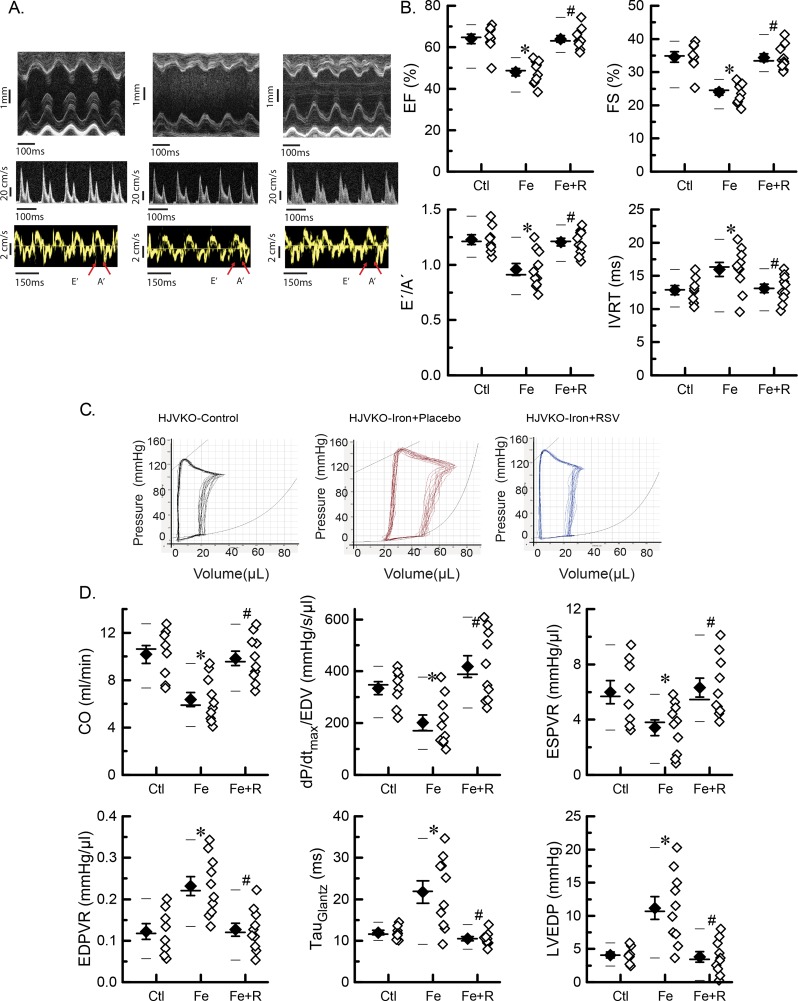
Cardiac function in advanced iron-overload cardiomyopathy (**A**) Representative images of M-mode (top panel), transmitral filling pattern (middle panel), and tissue Doppler (bottom panel). (**B**) Quantitative assessment of cardiac systolic and diastolic functions by echocardiography: EF, fractional shortening (FS), E′/A′ ratio, and isovolumic relaxation time (IVRT). (**C**) Representative PV loop traces. (**D**) Quantitative assessment of cardiac systolic and diastolic functions by PV loop analysis: cardiac output (CO), preload-adjusted contractility (dP/dt_max_/EDV), end-systolic PV relationship (ESPVR), end-diastolic PV relationship (EDPVR), isovolumic relaxation constant (*τ*, Glantz), and LV end-diastolic pressure (LVEDP). *n*=8 for the control group, *n*=10 for the iron + placebo group, and *n*=10 for the iron + RSV group. *P<0.05 compared with the control group; ^#^*P*<0.05 compared with the iron group. Abbreviations: Ctl, HJV control; Fe, iron diet; Fe + R, iron diet + resveratrol.

**Figure 3:**
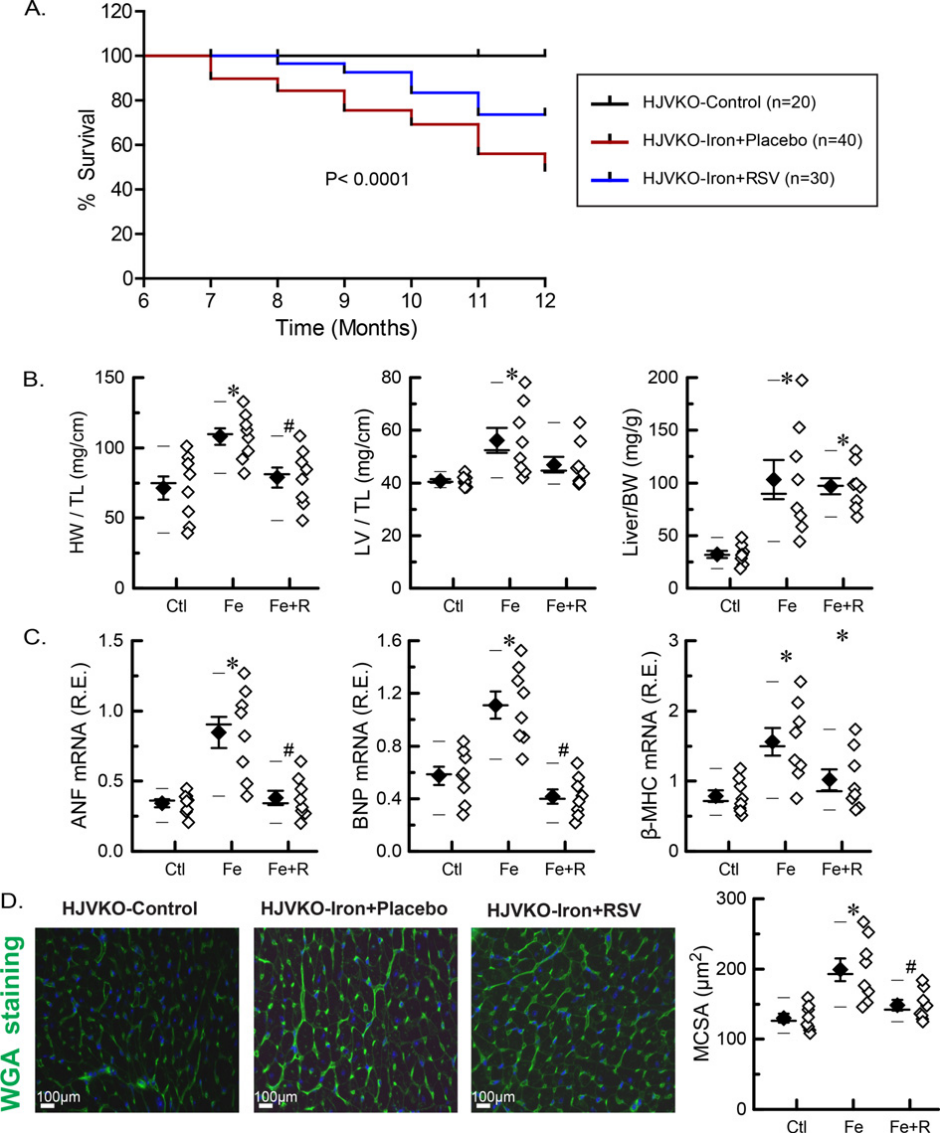
Survival and pathological hypertrophy in advanced iron-overload cardiomyopathy (**A**) Kaplan–Meier survival curves for control (HJVKO-control), iron overload (HJVKO-iron + placebo), and resveratrol rescue (HJVKO-iron + RSV). (**B**) Heart (HW), LV, and hepatic (liver) weights in control, iron overload, and after resveratrol therapy adjusted to tibia length (TL) or body weight (BW). (**C**) Expression levels of heart failure markers in control, iron overload, and after resveratrol therapy: ANF, BNP, and β-MHC. (**D**) Wheat germ agglutinin (WGA) staining: representative images and quantitation of myocardial cross-sectional area (MCSA) in control, iron overload, and after resveratrol therapy. *n*=8 hearts per group (B–D).*P<0.05 compared with the control group; ^#^*P*<0.05 compared with the iron group. Abbreviations: Ctl, HJV control; Fe, iron diet; Fe + R, iron diet + resveratrol.

The non-invasive echocardiographic assessment showed the presence of systolic and diastolic dysfunction in aged iron-overloaded HJVKO mice illustrated by representative M-mode, transmitral filling pattern, and tissue Doppler images characterized by lowered ejection fraction (EF) and fractional shortening, decreased E-prime to A-prime (E′/A′) ratio and E/E′ ratios, and prolonged isovolumic relaxation time (Figure 2A,B and Table 1). Blood pressure was not affected by either iron or resveratrol. We next performed invasive PV analysis to provide load-independent measures of LV performance. Invasive hemodynamics as illustrated by representative PV loops (Figure 2C) and quantitation (Figure 2D) confirmed marked systolic dysfunction with decreased contractile indices coupled with diastolic dysfunction characterized by increased myocardial stiffness and impaired relaxation. Resveratrol supplementation provided salutary therapeutic benefits and was associated with improved diastolic and systolic function based on echocardiography and hemodynamic analyses (Figure 2A–D). Progressive iron overload in HJVKO mice lowered survival by ~50% relative to control mice (Figure 3A) with the development of pathological hypertrophy as illustrated by morphometric assessment (Figure 3B), up-regulation in the expression of disease markers, atrial natriuretic factor (ANF), brain natriuretic peptide (BNP), and β-myosin heavy chain (β-MHC) (Figure 3C), and increased cardiomyocyte cross-sectional area (Figure 3D). Survival was improved (Figure 3A) and pathological hypertrophy was normalized by resveratrol treatment (Figure 3C,D). These results clearly demonstrate that ageing coupled with iron overload results in a severe cardiomyopathy and reduced survival, and resveratrol therapy rescued this phenotype at an advanced stage of the disease.

**Table 1:** Echocardiographic assessment of cardiac function and blood pressure in male HJVKO mice at 1 year of age

	HJVKO + vehicle	HJVKO + iron + placebo	HJVKO + iron + resveratrol
*n*	8	11	9
HR (echo) (bpm)	446 ± 13 (0.082)	433 ± 14 (0.107)	410 ± 11 (0.080)
E-wave (mm/s)	670 ± 47 (0.198)	682 ± 36 (0.175)	632 ± 18 (0.085)
A-wave (mm/s)	490 ± 45 (0.260)	437 ± 30 (0.228)	372 ± 33 (0.266)
E/A ratio	1.57 ± 0.10 (0.180)	1.49 ± 0.15 (0.334)	1.80 ± 0.20 (0.333)
DT (ms)	24.9 ± 1.0 (0.114)	32.8 ± 2.1* (0.216)	26.7 ± 2.2 (0.247)
EWDR (mm/s^2^)	27.4 ± 1.8 (0.184)	21.9 ± 2.2 (0.330)	24.7 ± 2.0 (0.244)
E′ (mm/s)	24.6 ± 1.3 (0.153)	20.1 ± 1.6 (0.264)	24.5 ± 1.6 (0.196)
A′ (mm/s)	20.1 ± 1.5 (0.211)	26.5 ± 1.5* (0.188)	20.4 ± 1.4^†^ (0.200)
E/E′ ratio	27.2 ± 2.5 (0.258)	33.9 ± 2.2 (0.212)	26.4 ± 1.6^†^ (0.176)
LA size (mm)	1.92 ± 0.08 (0.118)	2.20 ± 0.06* (0.090)	1.79 ± 0.09^^†^^ (0.151)
LVEDD (mm)	3.94 ± 0.12 (0.086)	4.12 ± 0.08 (0.064)	3.73 ± 0.09^†^ (0.072)
LVESD (mm)	2.59 ± 0.13 (0.142)	3.13 ± 0.08* (0.085)	2.45 ± 0.07^†^ (0.086)
VCFc (circ/s)	7.1 ± 0.4 (0.159)	5.1 ± 0.2* (0.130)	7.1 ± 0.2^†^ (0.101)
LVPWT (mm)	0.76 ± 0.03 (0.112)	0.93 ± 0.05* (0.178)	0.83 ± 0.04 (0.145)
HR (BP) (bmp)	425 ± 13 (0.084)	426 ± 16 (0.124)	407 ± 8 (0.060)
SBP (mmHg)	141 ± 4 (0.080)	139 ± 6 (0.148)	141 ± 5 (0.100)
DBP (mmHg)	93.3 ± 2.3 (0.070)	95.0 ± 3.3 (0.115)	96.0 ± 3.1 (0.097)
MAP (mmHg)	125 ± 3.3 (0.075)	125 ± 5.1 (0.136)	126 ± 4.1 (0.098)

Data are presented as mean ± S.E.M. (coefficient of variation). *P<0.05 compared with vehicle group, ^†^*P*<0.05 compared with iron group. Abbreviations: A, atrial transmitral filling wave; A′, tissue Doppler due to atrial contraction; DBP, diastolic blood pressure; DT, deceleration time; E, early transmitral filling wave; E′, early tissue Doppler velocity; EWDR, E-wave deceleration rate; HR, heart rate; LA, left atrium; LVEDD, LV end-diastolic dimension; LVESD, LV end-systolic dimension; LVPWT, LV posterior wall thickness; MAP, mean arterial pressure; SBP, systolic blood pressure; VCFc, velocity of circumferential fiber shortening.

### Resveratrol ameliorated myocardial fibrosis and Ca^2+^ cycling defects in iron-overload cardiomyopathy

**Figure 4:**
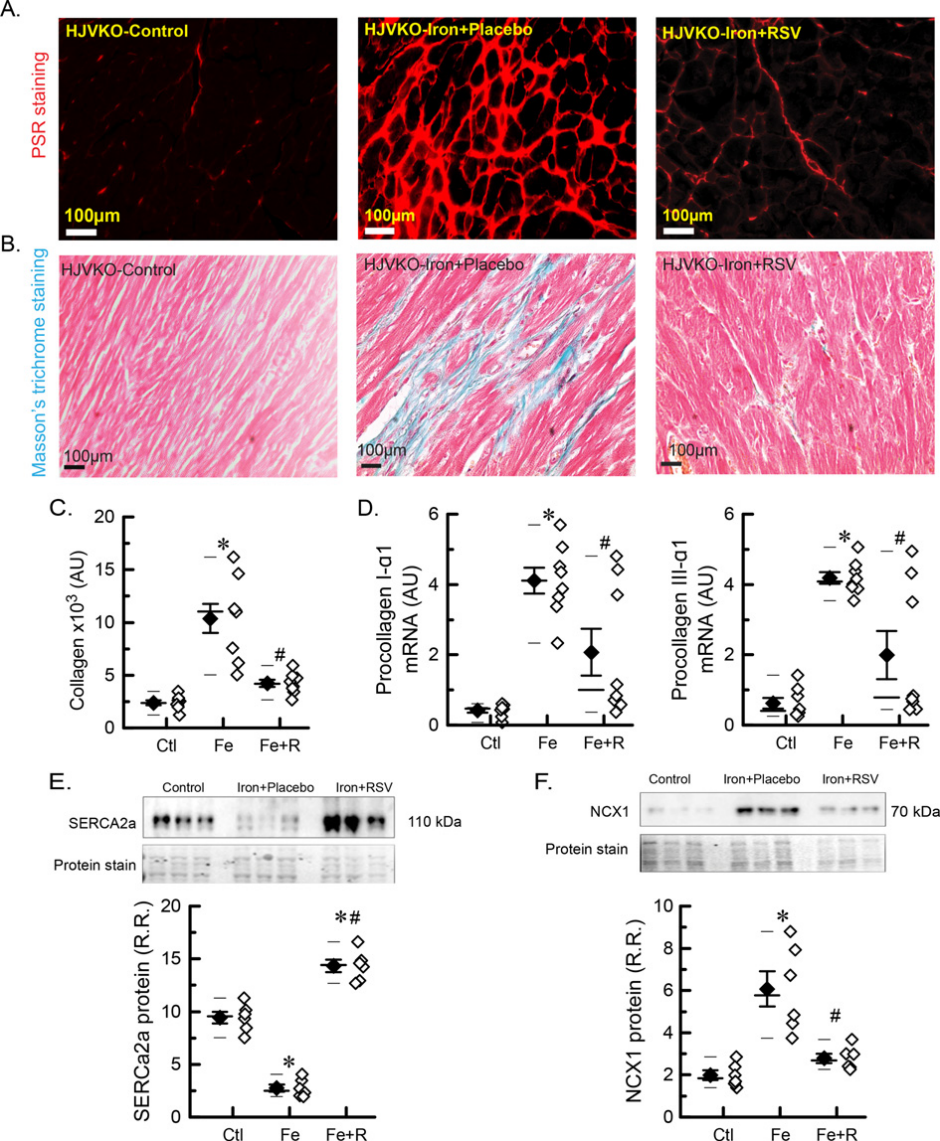
Myocardial fibrosis and Ca^2+^ cycling proteins in advanced iron-overload cardiomyopathy (**A**) Representative images of PSR staining. (**B**) Representative images of Masson’s trichrome staining. (**C**) Quantitation of PSR staining to assess fibrosis levels (*n*=4 hearts; two sections per heart). (**D**) Expression levels of myocardial procollagen Iα1 and procollagen IIIα1 (*n*=8 hearts). (**E**) Western blot analysis and quantitation of myocardial SERCa2a protein levels. (**F**) Western blot analysis and quantitation of myocardial NCX1 protein levels. *n*=6 hearts for Western blot analysis (E,F).*P<0.05 compared with the control group; ^#^*P*<0.05 compared with the iron group. Abbreviations: AU, arbitrary unit; Ctl, HJV control; Fe, iron diet; Fe + R, iron diet + resveratrol; R.R., relative ratio.

We next assessed myocardial fibrosis and Ca^2+^ cycling proteins, which are key determinants of diastolic and systolic dysfunction [20,30,31]. PSR and Masson’s trichrome staining revealed marked interstitial fibrosis in iron-overloaded hearts (Figure 4A,B; quantitation of the PSR staining, Figure 4C). Myocardial expression of *procollagen I and III* mRNA was significantly increased in aged iron-overloaded mice (Figure 4D) in the absence of tissue inflammation based on expression of inflammatory markers (Supplementary Table S2) and immunostaining for neutrophils and macrophages (Supplementary Figures S2 and S3). Resveratrol therapy resulted in marked suppression of myocardial fibrosis and ameliorated overexpression of the profibrotic myocardial genes (Figure 4A–D). Defective SERCa2a function impairing Ca^2+^ uptake and leading to abnormal Ca^2+^ cycling is a fundamental mechanism in the progression of systolic heart failure [31,32]. Iron-overloaded hearts showed a marked reduction in SERCa2a protein levels with a compensatory increase in NCX1 levels (Figure 4E,F). Resveratrol therapy resulted in a significant increase in SERCa2a protein levels accompanied by normalization of NCX1 levels (Figure 4E,F). Advanced iron-overload cardiomyopathy is associated with increased myocardial fibrosis and down-regulation of the SERCa2a system, which were rescued by resveratrol therapy.

### Iron-induced oxidative stress is moderated by resveratrol therapy

**Figure 5:**
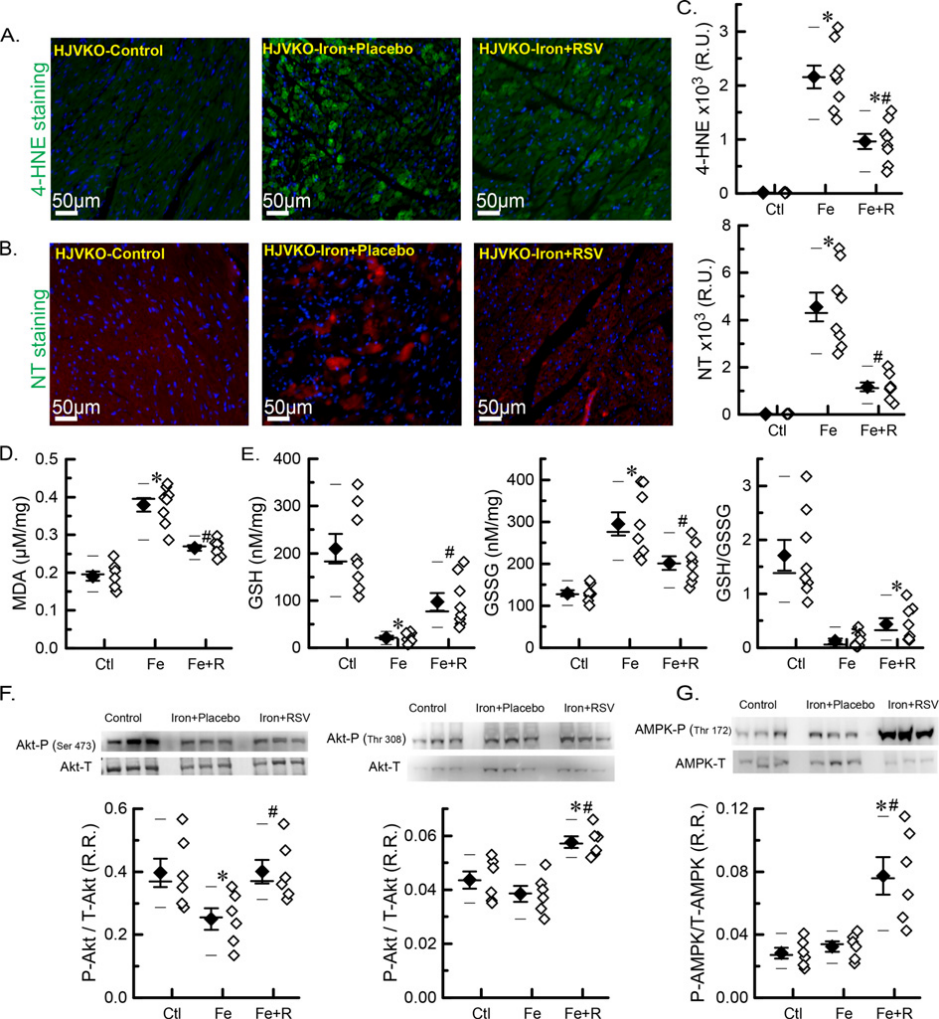
Myocardial oxidative stress and metabolic signaling in advanced iron-overload cardiomyopathy (**A**) Representative images of immunofluorescence staining for 4-HNE. (**B**) Representative images of immunofluorescence staining for NT. (**C**) Quantitations of 4-HNE and NT stainings (*n*=4 hearts; two sections per heart). (**D**) Levels of myocardial lipid peroxidation product, MDA. (**E**) Levels of GSH and GSSG, as well as GSH/GSSG ratio. (**F**) Western blot analysis and quantitation of p-Ser^473^ Akt and p-Thr^308^ Akt phosphorylation ratios. (**G**) Western blot analysis and quantitation of p-Thr^172^-AMPK phosphorylation ratio. *n*=8 for biochemical analysis (D,E); *n*=6 for Western blot analysis (F,G). *P<0.05 compared with the control group; ^#^*P*<0.05 compared with the iron group. Abbreviations: Ctl, HJV control; Fe, iron diet; Fe + R, iron diet + resveratrol; R.R., relative ratio; R.U., relative unit.

Iron overload stimulates Fenton chemistry leading to the generation of oxidative free radicals and damage including lipid peroxidation [4,15,16,19,33]. We characterized the myocardial oxidative stress in the aged iron-overloaded hearts and evaluated the beneficial effects of resveratrol. We found increased 4-HNE lipid peroxidation adducts and peroxynitrite formation based on NT immunostaining (Figure 5A–C) and higher levels of MDA, a product of lipid peroxidation (Figure 5D) in the iron-overloaded myocardium. Iron overload also resulted in a marked lowering of a key non-enzymatic intrinsic antioxidant, GSH, and increased accumulation of GSSG (Figure 5E). Resveratrol therapy markedly suppressed the pro-oxidative state in iron-overloaded hearts resulting in lower levels of 4-HNE and NT immunostaining (Figure 5C), as well as lower levels of MDA (Figure 5D) and closer to normal levels of glutathiones (Figure 5E). Oxidative stress is known to activate metabolic signaling pathways. We found that Ser^473^ phosphorylation of Akt was significantly reduced in iron-overloaded hearts while resveratrol treatment normalized Ser^473^ phosphorylation and increased phosphorylation of the Thr^308^ residue of Akt (Figure 5F) and Thr^172^ residue of AMPK (Figure 5G). Our results clearly demonstrate that iron induces oxidative stress, and the oxidative stress and pathological metabolic signaling were ameliorated by resveratrol therapy.

## Discussion

Iron-overload cardiomyopathy is a major cause of heart failure in patients with primary hemochromatosis and secondary iron overload [3–5,10,14,34]. However, current therapeutic approaches and understanding of the details of pathophysiological mechanisms are limited, in part, by the lack of a preclinical model, which can closely recapitulate iron-overload cardiomyopathy in patients [5,10]. Recently, we reported that HJVKO mice develop diastolic dysfunction, but systolic function was not affected by the age of 6 months in response to 5 months of high-iron diet [20]. Diastolic dysfunction was accompanied by oxidative stress and developed due to increased fibrosis, and possibly elevated NCX1 and reduced SERCa2a protein levels (demonstrated for WT model with injected iron dextran, but was not shown for HJVKO with iron diet) [20]. In this study, the high-iron diet was administered up to 1-year age to ensure sufficient time for iron injury to develop. The extended iron exposure modestly increased myocardial iron levels from 3 mg/g at 6 months [20] to 3.5 mg/g at 1 year as reported in the current study. However, the extended iron exposure resulted in a more severe phenotype characterized by both diastolic and systolic dysfunctions, suggesting that the latest approach is considerably more accurate in simulating iron-overload cardiomyopathy. In the present study, we were able to directly confirm that diastolic dysfunction arises from both: (i) passive component (an increased cardiac tissue stiffness due to fibrosis), and (ii) active component (dysregulation of Ca^2+^ handling proteins that impairs relaxation). Using this improved and more adequate model of iron-overload cardiomyopathy, we explored the therapeutic effects of resveratrol in prevention and possible reversion of development of iron-overload cardiomyopathy and found that myocardial oxidative stress, fibrosis, and Ca^2+^ cycling defects linked to the diastolic and systolic dysfunction can be ameliorated with resveratrol supplementation. Based on this, we propose that resveratrol can be a potential therapeutic intervention to reduce and/or revert progression of iron-overload cardiomyopathy at the advanced stage of iron overload.

A preclinical murine model of advanced cardiomyopathy (HJVKO male mice with high-iron diet) presented in the present study has iron levels comparable with iron levels in patients with iron-overload cardiomyopathy: myocardial iron levels in our murine model are ~3.5 mg/g LV weight and myocardial iron levels in patients with iron-overload cardiomyopathy and heart failure are 3–9 mg/g LV weight [35]. As we have previously established, resveratrol supplementation in HJVKO mice treated with a normal diet did not alter cardiac structure and function [20]. Surprisingly, resveratrol markedly improved cardiac function and rescued the advanced iron-overload cardiomyopathy in aged HJVKO mice without affecting myocardial iron levels. Resveratrol therapy prevented deterioration and possibly improved systolic and diastolic function based on both echocardiographic and hemodynamic techniques. These results are consistent with the ability of resveratrol to prevent diastolic dysfunction and heart disease in murine models with a less developed iron-overload cardiomyopathy characterized exclusively by diastolic dysfunction with preserved EF [20].

Myocardial remodeling, pathological hypertrophy, and increased expression of fetal genes are the major contributors to the progression of advanced heart failure [23,26]. Resveratrol therapy effectively moderated adverse myocardial remodeling and the expression of fetal gene programming in the myocardium: both 6-month [20] and 1-year iron groups had elevated HF markers, whereas resveratrol-treated group (1-year iron with 3-month resveratrol) had improved HF marker expression pattern. Many lines of evidence have shown that resveratrol inhibits pathological hypertrophy and myocardial remodeling, predominantly by activating AMPK amongst other cardioprotective and metabolic benefits [23,36–40]. We also observed that resveratrol treatment increased the phosphorylation of AMPK. Myocardial fibrosis is a key driver in the progression of advanced heart failure and mediates diastolic dysfunction [20,41]. By 6 months of exposure to iron, myocardial collagen content is already substantially increased [20]. Initiation of resveratrol therapy at 9 months substantially reduced myocardial fibrosis leading to normalized ventricular stiffness and LV end-diastolic pressure, and is consistent with resveratrol ability to normalize the expression of matrix metalloproteases in pressure-overload heart failure [23]. These results are consistent with resveratrol ability to suppress the profibrotic effects of iron on murine and human cardiofibroblasts [20]. Advanced heart failure is also associated with significant reduction in *SERCA2a* mRNA and protein levels leading to defective Ca^2+^ handling [31,42]. We found decreased SERCa2a protein levels as a result of iron-overload cardiomyopathy with a compensatory increase in the NCX1. Since SERCa2a and NCX1 protein levels were reduced and elevated, respectively, at 6 months of iron [20], resveratrol treatment from 9 months to 1 year of iron administration up-regulated SERCa2a and reduced NCX1 protein levels in association with improved cardiac function that can be viewed as rescuing action of resveratrol.

Iron-induced oxidative stress is a key driver in the pathogenesis of iron-overload cardiomyopathy [16,19]. Antioxidants, *N*-acetyl cysteine and taurine, have been shown to rescue iron-mediated injury in the brain [43] and heart [16,44]. Combining antioxidant with iron chelation causes a greater improvement than use of chelation agents by themselves [43,44]. However, in the settings of acute iron intoxication, *N*-acetyl cysteine can have deleterious effects [45]. Unlike other antioxidants, resveratrol can improve cardiac metabolism [23], protecting Ca^2+^ cycling proteins [46], and activate prosurvival signaling pathways [20,47]. We also characterized iron-induced oxidative stress in advanced iron-overload murine models. Consistent with our previous reports showing that myocardial iron overload is associated with increased oxidative stress [16,20,48], we also observed elevated oxidative stress characterized by reduced glutathione levels and increased lipid peroxidation in advanced iron-overload cardiomyopathy. Furthermore, lipid peroxidation end products are toxic and cause cellular dysfunction and interfere with the excitation–contraction coupling properties of heart [48]. Consistent with this, resveratrol administration significantly ameliorated oxidative stress and lipid peroxidation. Iron overload can result in diabetes, which was not seen in our model, since aged iron-overloaded HJVKO mice showed no overt changes in body composition and had normal glucose tolerance [29]. Preclinical studies with animal models showed significant beneficial effects of resveratrol such as antioxidant and metabolic effects as well as an ability to activate prosurvival signaling pathways [20,23,46,47,49–52]. While translating the beneficial effects of resveratrol seen in our preclinical model into clinical use will be challenging due to the low bioavailability, variability in patient responses, and rapid metabolism of the parent compound [53,54], the potential clinical impact at an international scale warrants future clinical trials.

In summary, aged iron-overloaded HJVKO murine model showed clear evidence of advanced iron-overload cardiomyopathy. Resveratrol supplementation rescued the progression of iron-induced oxidative damage, improved SERCa2a levels, and reversed pathological hypertrophy and fibrosis leading to normalized cardiac function in iron-overloaded mice. Several studies have confirmed the pleiotropic beneficial effects of resveratrol without any toxic side effects and we propose that dietary intake of resveratrol represents a useful intervention to revert progression of iron-induced cardiac dysfunction, thereby potentially reducing the global clinical burden of iron-overload cardiomyopathy.

## Supporting information

**Supplemental Figure 1:** Body composition in HJVKO mice with advanced iron-overload cardiomyopathy. **A.** Body weight. **B.** Total fat. **C.** Fat mass to body weight ratio. **D.** Lean mass to body weight ratio. **E.** Total body water. Ctl=HJV control, Fe=iron diet, Fe+R=iron diet + resveratrol; values are the mean±SEM of n=5 hearts in each group.

**Supplemental Figure 2:** Neutrophil immunostaining of cardiac sections of HJVKO mice with advanced iron-overload cardiomyopathy. **A.** Representative images of neutrophil (Ly-6B.2 alloantigen) immunostaining. **B.** Neutrophil count derived from neutrophil immunostaining of cardiac sections. Ctl=HJV control, Fe=iron diet, Fe+R=iron diet + resveratrol; values are the mean±SEM of n=6 (3 hearts; 2 sections per heart) for the control, n=8 (4 hearts; 2 sections per heart) for the iron+placebo group, n=8 (4 hearts; 2 sections per heart) for the iron+RSV group, and n=8 (4 hearts; 2 sections per heart) for the positive control (3-day MI); ND=not detected; ^*^p<0.05 compared with the control group; ^#^p<0.05 compared with the iron group.

**Supplemental Figure 3:** Macrophage immunostaining of cardiac sections of HJVKO mice with advanced iron-overload cardiomyopathy. **A.** Representative images of macrophage (F4/80) immunostaining. **B.** Macrophage count derived from macrophage immunostaining of cardiac sections. Ctl=HJV control, Fe=iron diet, Fe+R=iron diet + resveratrol; values are the mean±SEM of n=6 (3 hearts; 2 sections per heart) for the control, n=8 (4 hearts; 2 sections per heart) for the iron+placebo group, n=8 (4 hearts; 2 sections per heart) for the iron+RSV group, and n=8 (4 hearts; 2 sections per heart) for the positive control (3-day MI); ND=not detected; ^*^p<0.05 compared with the control group; ^#^p<0.05 compared with the iron group.

**Supplemental Table 1.** List of Taqman Primers and Probes

**Supplemental Table 2.** Myocardial gene expression analysis of inflammatory cytokines in hemojuvelin knockout mice at 1 year of age

## Abbreviations

Akt: protein kinase B
AMPK: AMP-activated protein kinase
E′/A′: E-prime to A-prime
EDV: end-diastolic volume
EF: ejection fraction
HF: heart failure
HJV: hemojuvelin
HJVKO: HJV knockout
HRP: horseradish peroxidase
LV: left ventricular
MDA: malondialdehyde
NCX1: sodium–calcium exchanger
NT: nitrotyrosine
OCT: optimum temperature cutting medium
PSR: Picrosirius Red
PV: pressure–volume
SERCa2a: myocardial sarcoplasmic reticulum Ca^2+^ ATPase
WT: wild-type
4-HNE: 4-hydroxynonenal
