# Supplementary material for: Advanced iron-overload cardiomyopathy in a genetic murine model is rescued by resveratrol therapy
Source: Biosci Rep. 2018 Jan 10;38(1):BSR20171302. doi: 10.1042/BSR20171302 (PMC6435471; doi:10.1042/BSR20171302)
Supplement: Supplementary file 1 [file bsr20171302_Supp1.pdf]

Advanced iron-overload cardiomyopathy in a genetic murine model is rescued by resveratrol therapy

by

Subhash K. Das[1,2,3], Pavel Zhabyeyev[1,2,3] Ratnadeep Basu[1,2,3], Vaibhav B. Patel[1,2,3], Jason R. B. Dyck[3,5], Zamaneh Kassiri[2,3,4], and Gavin Y. Oudit[1,2,3]

[1]Division of Cardiology, Department of Medicine, [2]Mazankowski Alberta Heart Institute, [3]Cardiovascular Research Centre, [4]Department of Physiology, University of Alberta, [5]Departments of Pediatrics and Pharmacology, University of Alberta, Edmonton, Canada

Supplemental Table 1: List of Taqman Primers and Probes

Gene	Type	Sequence
ANF	Forward:<br>Reverse:<br>Probe:	5'-GGA GGA GAA GAT GCC GGT AGA-3'<br>5'-GCT TCC TCA GTC TGC TCA CTC A-3'<br>5'-FAM-TGA GGT CAT GCC CCC GCA GG-TAMRA-3’
BNP	Forward:<br>Reverse:<br>Probe:	5'-CTG CTG GAG CTG ATA AGA GA-3'<br>5'-TGC CCA AAG CAG CTT GAG AT-3'<br>5'-FAM-CTC AAG GCA GCA CCC TCC GGG-TAMRA-3'
β- MHC	Forward:<br>Reverse:<br>Probe:	5'-GTGCCA AGG GCC TGA ATG AG-3'<br>5'-GCA AAG GCT CCA GGT CTG A-3'<br>5'-FAM-ATC TTG TGC TAC CCA GCT CTA A-TAMRA-3'
Pro-Collagen-I	Forward:<br>Reverse:<br>Probe:	5’- CTTCACCTACAGCACCCTTGTG-3’<br>5’-TGACTGTCTTGCCCCAAGTTC-3’<br>5’-FAM-CTGCACGAGTCACACC-TAMRA-3'
Pro-Collagen-III	Forward:<br>Reverse:<br>Probe	5’- TGTCCTTTGCGATGACATAATCTG -3’<br>5’- AATGGGATCTCTGGGTTGGG-3’<br>5’-FAM- ATGAGGAGCCACTAGACT-TAMRA-3'
IL-6	Forward:<br>Reverse:<br>Probe:	5'-ACAACCACGGCCTTCCCTACTT-3’<br>5'-CACGATTTCCCAGAGAACATGTG-3’<br>5'-FAM-TTCACAGAGGATACCACTCCCAACAGACCT-TAMRA-3’
IL-1β	Forward:<br>Reverse:<br>Probe:	5'-AACCTGCTGGTGTGTGACGTTC-3’<br>5'-CAGCACGAGGCTTTTTTGTTGT-3’<br>5'- FAM-TTAGACAGCTGCACTACAGGCTCCGAGATG-TAMRA-3’
TNF-α	Forward:<br>Reverse:<br>Probe:	5'- ACAAGGCTGCCCCGACTAC-3’<br>5'- TTTCTCCTGGTATGAGATAGCAAATC-3’<br>5'-FAM-TGCTCCTCACCCACACCGTCAGC-TAMRA-3’
Trfc(Transferrin R)	premix	Mm00441941_m1*
HJV(Hemojuvelin)	Premix	Mm00510148_s1
FPN1(Ferroportin)	Premix	Mm00489837_m1*
HAMP1(Hepcidin1)	Premix	Mm00519025_m1
Ftl1(Ferritin-L)	Premix	Mm03030144_g1
Fth1(Ferritin-H)	Premix	Mm00085707_g1
18S rRNA	Premix	Mm03928990_g1*

Supplemental Table 2. Myocardial gene expression analysis of inflammatory cytokines in hemojuvelin knockout mice at 1 year of age

	HJVKO+Vehicle	HJVKO+Iron+Placebo	HJVKO+Iron+Resveratrol
n	8	8	8
TNFα (R.E.)	3.12±0.24 (0.218)	2.67±0.31 (0.328)	3.72±0.37 (0.281)
IL-1β (R.E.)	1.69±0.26 (0.435)	1.89±0.38 (0.569)	1.58±0.34 (0.609)
IL-6 (R.E.)	7.46±1.03 (0.391)	8.01±1.45 (0.512)	6.22±1.26 (0.573)

Data are presented as Mean ± SEM (CV). CV = coefficient of variation. TNF = tumour necrosis factor alpha; IL-1β = interleukin 1 beta; IL-6 = interleukin 6; R.E.=relative expression.

Supplemental Figure Legends

Supplemental Figure 1. Body composition in HJVKO mice with advanced iron-overload cardiomyopathy. **A**. Body weight. **B**. Total fat. **C**. Fat mass to body weight ratio. **D**. Lean mass to body weight ratio. **E**. Total body water. Ctl=HJV control, Fe=iron diet, Fe+R=iron diet + resveratrol; values are the mean±SEM of n=5 hearts in each group.

Supplemental Figure 2. Neutrophil immunostaining of cardiac sections of HJVKO mice with advanced iron-overload cardiomyopathy. **A**. Representative images of neutrophil (Ly-6B.2 alloantigen) immunostaining. **B**. Neutrophil count derived from neutrophil immunostaining of cardiac sections. Ctl=HJV control, Fe=iron diet, Fe+R=iron diet + resveratrol; values are the mean±SEM of n=6 (3 hearts; 2 sections per heart) for the control, n=8 (4 hearts; 2 sections per heart) for the iron+placebo group, n=8 (4 hearts; 2 sections per heart) for the iron+RSV group, and n=8 (4 hearts; 2 sections per heart) for the positive control (3-day MI); ND=not detected; *$p<0.05$ compared with the control group; $^{\#}p<0.05$ compared with the iron group.

Supplemental Figure 3. Macrophage immunostaining of cardiac sections of HJVKO mice with advanced iron-overload cardiomyopathy. **A**. Representative images of macrophage (F4/80) immunostaining. **B**. Macrophage count derived from macrophage immunostaining of cardiac sections. Ctl=HJV control, Fe=iron diet, Fe+R=iron diet + resveratrol; values are the mean±SEM of n=6 (3 hearts; 2 sections per heart) for the control, n=8 (4 hearts; 2 sections per heart) for the iron+placebo group, n=8 (4 hearts; 2 sections per heart) for the iron+RSV group, and n=8 (4 hearts; 2 sections per heart) for the positive control (3-day MI); ND=not detected; *$p<0.05$ compared with the control group; $^{\#}p<0.05$ compared with the iron group.

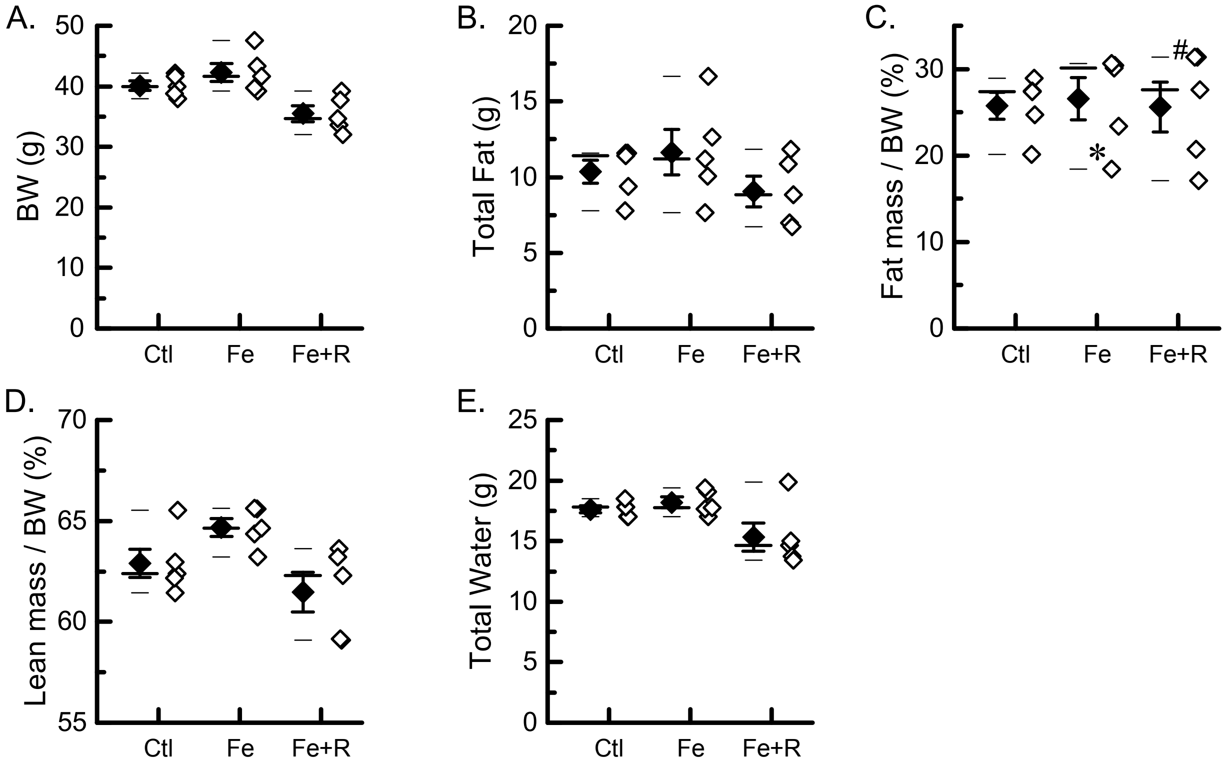

Das SK. *et al.* Supplemental Figure 1

A.

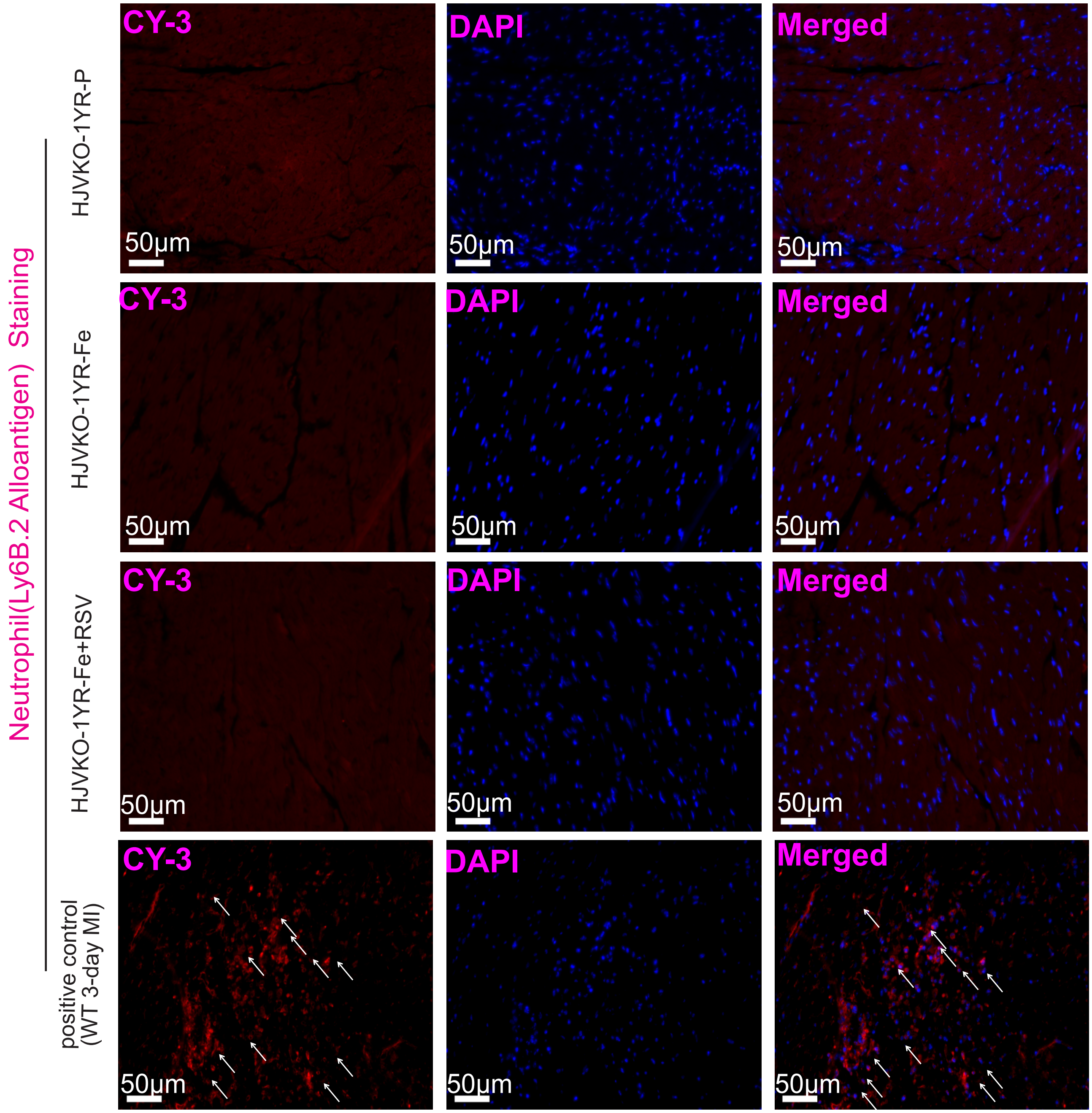

B.

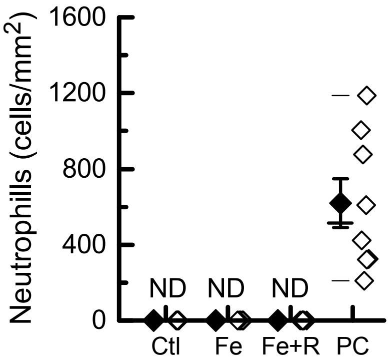

Das SK. *et al.* Supplemental Figure 2

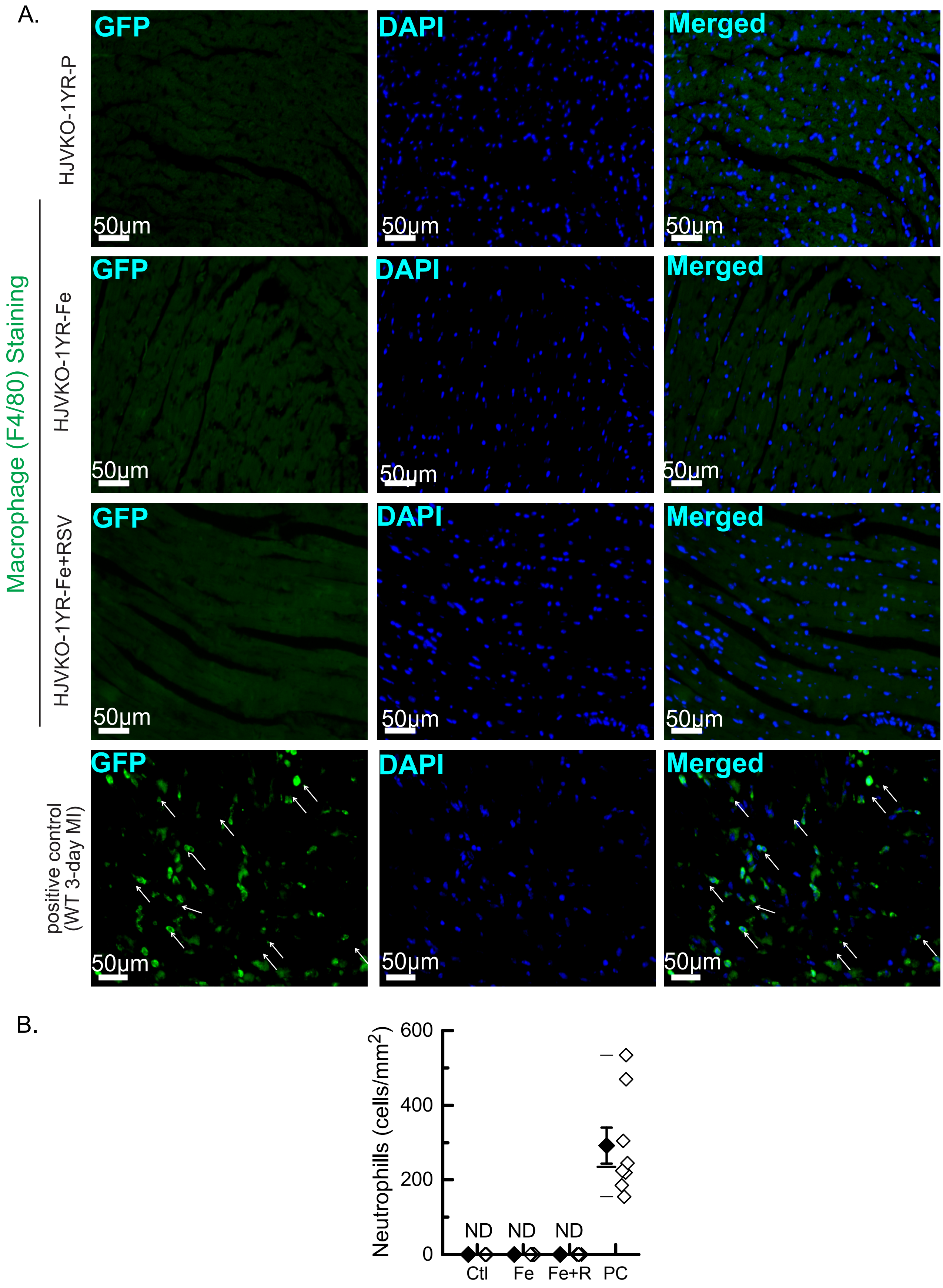

Das SK. *et al.* Supplemental Figure 3
